# Experimental data on bacterial abundance and morphological changes in copepod carcasses during their decomposition (*in vitro*)

**DOI:** 10.1016/j.dib.2020.105563

**Published:** 2020-04-22

**Authors:** Nikolay V. Lobus, Elena M. Bezzubova, Daria A. Litvinyuk

**Affiliations:** a Shirshov Institute of Oceanology of the Russian Academy of Sciences, Moscow, Russian Federation; b A.O. Kovalevsky Institute of Biology of the Southern Seas of the Russian Academy of Sciences, Sevastopol, Russian Federation

**Keywords:** Dead zooplankton, Decomposition of copepod carcasses, Bacterial activity, Arctic environment

## Abstract

The biogeochemical role of zooplankton in the ocean is determined not only by life-long accumulation of chemical elements from the environment, but also by post-mortal transformation of carcasses chemical composition. The contribution of zooplankton carcasses to vertical flux of major and trace elements depends on sedimentation and remineralization rates of detrital particles. Carcasses decomposition rate during sinking from the upper to the deeper water layers determines the rapid recycling of chemical elements and depends on ambient temperature and microbial activity. This data set summarizes 21-day experiment in microcosms that simulates temperature conditions in the Arctic environment. The data show slow decomposition of copepod carcasses compared with initial material on days 14–21 of the experiment. In addition to visual evidence, we provide data on changes in bacterial abundance and biomass during the whole experimental period.

Specifications Table

**Table:** 

Subject	Oceanography
Specific subject area	Biogeochemistry, Biogeochemical cycles of elements, Ocean ecology
Type of data	Tables, Images, Figures
How data were acquired	Microscope (Leica DM5000 B for estimation of bacteria, Nikon Eclipse TS-100 equipped with digital camera Nikon D5100 for zooplankton analysis), ImageScope Colour software.
	Statistical analyzes were performed in MS Excel 2010 and Statgraphics Plus Software.
Data format	Raw, Analyzed
Parameters for data collection	Microcosm experiment was performed with environmental samples of sea water and zooplankton carcasses. Microcosms were incubated in thermostat for 21 days (sampling days 0, 1, 3, 5, 7, 14, 21) at a temperature ranged from −1.2 to −0.9 °C. 18 microcosms (3 frequencies for each experimental day 1–21) were enriched on Day 0 with zooplankton. Six others received no manipulation and served as control.
Description of data collection	Samplings of three experimental and one control microcosms were performed every experimental day at the same time.
	Zooplankton carcasses were visually analysed and photographed under an inverted microscope equipped with digital camera. The analysis was focused on postmortem changes and the most typical signs of decomposition of *Calanus* spp. carcasses.
	Bacterial abundance was obtained with epifluorescence microscopy. 20 fields and a total of >200 bacteria were counted for each filter. The bacterial biomass carbon was estimated based on bacterial cell volume.
Data source location	Sampling was carried out from the Kara Sea (Russia) during the 69^rd^ cruise of *R/V Academik Mstislav Keldysh*.
	Zooplankton was collected at the 5586/2 station (N 73.10° E 63.32°)
	Water sample was taken at 5582/2 station (N 70.36° E 58.20°) using Niskin bottles.
	The experiment was performed in Shirshov Institute of Oceanology of the Russian Academy of Sciences, Moscow, Russia.
Data accessibility	Data on bacterial abundance and morphological changes in copepod carcasses are provided with this article.

## Value of the data

• Experimentally obtained data demonstrate the microbial abundance associated with the decomposition process of zooplankton carcasses.
• This dataset could be used for estimating the rate of zooplankton carcasses decomposition, as well as for experimental studying of post-mortal changes in chemical composition of organisms under Arctic environmental conditions.
• A comparative analysis of the data is important for understanding the role of zooplankton and its carcasses in biogeochemical processes of major and trace elements distribution.

## 1 Data description

Thermostat temperature range during the experiment is presented in Table 1. The temperature changes did not exceed 0.3 °C.

**Table 1:** Thermostat temperature range during the experiment.

Date (dd.mm.yyyy)	Day of sampling	Temperature, °C
04.03.2019	0	−1.2
05.03.2019	1	−1.1
06.03.2019		−1.1
07.03.2019	3	−1.1
09.03.2019	5	−1.2
11.03.2019	7	−0.9
12.03.2019		−1.0
13.03.2019		−1.2
14.03.2019		−1.2
15.03.2019		−1.1
18.03.2019	14	−1.1
19.03.2019		−1.0
20.03.2019		−0.9
21.03.2019		−1.1
22.03.2019		−1.2
23.03.2019		−1.0
24.03.2019		−1.1
25.03.2019	21	−1.1

The dataset of measured bacterial parameters in microcosm water is tabulated in Table 2.

**Table 2:** Bacterial abundance (10^6^ cells × ml^−1^), biomass (mgC × *m*^−3^). The mean values are presented with standard deviation (±).

Day	Sample	Abundance, × 10^6^ cells ml^-1^	Mean abundance, × 10^6^ cells ml^-1^	Mean biomass, mgC × m ^−3^
0	Control	0.16 ± 0.019	0.16 ± 0.019	3.23 ± 0.40
1	Control	0.19 ± 0.016	0.19 ± 0.016	2.16 ± 0.18
	1.1	0.45 ± 0.046		
	1.2	0.15 ± 0.035	0.25 ± 0.160	1.41± 0.91
	1.3	0.14 ± 0.025		
3	Control	0.19 ± 0.023	0.19 ± 0.023	2.08 ± 0.25
	3.1	0.20 ± 0.021		
	3.2	0.15 ± 0.029	0.18 ± 0.029	2.63 ± 0.42
	3.3	0.18 ± 0.005		
5	Control	0.13 ± 0.001	0.13 ± 0.001	1.48 ± 0.01
	5.1	0.45 ± 0.009		
	5.2	0.34 ± 0.027	0.35 ± 0.079	4.81 ± 1.08
	5.3	0.28 ± 0.002		
7	Control	0.17 ± 0.007	0.17 ± 0.007	0.97 ± 0.04
	7.1	0.07 ± 0.009		
	7.2	0.19 ± 0.031	0.18 ± 0.093	1.78 ± 0.94
	7.3	0.27 ± 0.031		
14	Control	0.23 ± 0.002	0.23 ± 0.002	1.86 ± 0.01
	14.1	10.14 ± 0.540		
	14.2	23.11 ± 0.584	16.63 ± 7.50	74.37 ± 33.56
	14.3	Sample lost		
21	Control	0.28 ± 0.047	0.28 ± 0.047	2.68 ± 0.44
	21.1	28.74 ± 0.001		
	21.2	38.21 ± 1.169	34.36 ± 4.65	314.95 ± 42.75
	21.3	36.06 ± 2.878		

**Fig. 1:**
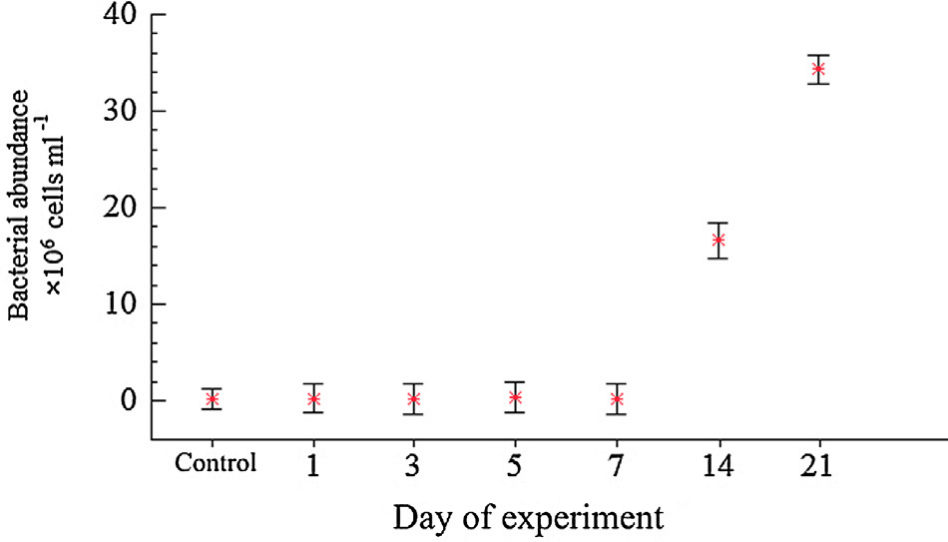
Changes of bacterial abundance during the microcosm experiment. Multiple comparisons were performed through 95% LSD intervals. Data are mean±SD of three replicates from Day 1 to Day 21. Control (no zooplankton addition) data are mean±SD of seven samples (1 replicate for each experimental day from 0 to 21).

Fig. 1 represents changes of bacterial abundance in water during carcasses decomposition. Differences between control and experiments were compared by Fisher's least-significance test (LSD; *p* = 0.05). Starting from initial abundance of 0.16 × 10^6^ cells ml^-1^ bacteria in water increased on Day 1 in the control and one of the experimental microcosms. Bacterial abundance in two other microcosms water decreased compared to Day 0. On Day 3, mean bacterial abundance in the experiments decreased while in the control it remained constant. Significant differences in bacterial abundance between control and experimental treatment were observed on Day 5. Bacterial abundance in the control microcosm decreased compared to Day 3 while the abundance in the experiments increased twice. On Day 7, bacterial abundance in all experimental microcosms decreased more than two times compared to Day 5. Density of heterotrophic bacteria in the control microcosm continuously increased from Day 7 until the end of the experiment. In the experimental microcosms the bacteria numbers increased rapidly from Day 14. Mean bacterial abundance increased almost a hundred times on Day 14 and more than two hundred times on Day 21 compared to initial day numbers. No significant differences in bacterial abundance were detected between control and experimental microcosms until Day 14 (*p* ≥ 0.05).

**Fig. 2:**
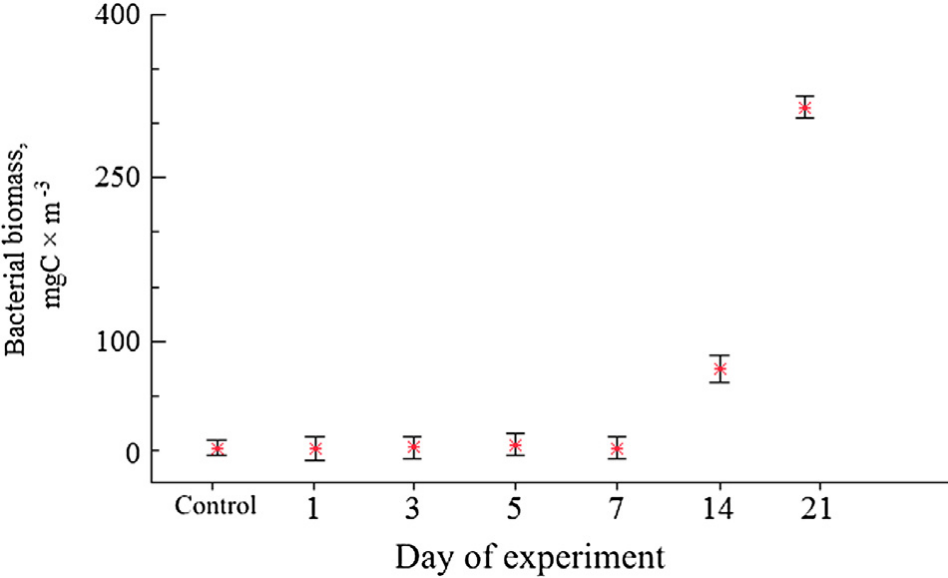
Temporal changes in bacterial biomass (means with 95% LSD intervals). Data are mean±SD of three replicates from Day 1 to Day 21. Control (no zooplankton addition) data are mean±SD of seven samples (1 replicate for each experimental day from 0 to 21).

**Fig. 3:**
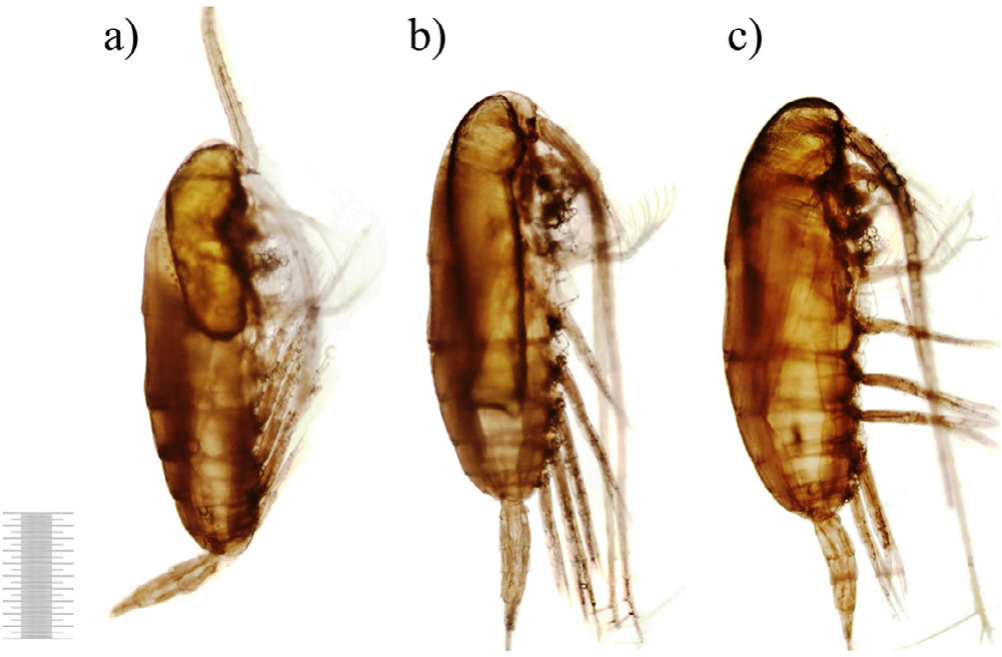
Copepod carcasses on Day 0 (1 mm scale).

Bacterial biomass dynamics in the experimental microcosms were similar to abundance changes (Fig. 2). In contrast, in the controls bacterial biomass numbers were lower than initial 3.23 ± 0.40 mgC × m^-3^ during all experimental days. In waters of experimental microcosms bacterial biomass first decreased (Day 1), then slightly increased until Day 5 and dropped on Day 7. After Day 7 value of biomass experienced an abrupt increase towards Day 14 and exceeded almost hundred times of the initial level on Day 21. From Day 14, there was a distinct and significant difference in bacterial biomass between control and experiments (*p* ≥ 0.05). Starting from Day 5 bacteria tended to granulate. On Day 14 large bacterial aggregates were already formed. The initial morphological condition of copepods carcasses is presented in the control, сopepod carcasses had integral external integuments without ruptures and carapace detachments (Fig. 3). The whole carcass was intensive yellow-brown in color that characterized living organisms, antennae and thoracic legs were homogeneously pigmented. The internal tissues were clearly visible; muscle fibers were firmly attached to the carapace. Very large oil sac remained present and typically elongated along the entire body (Fig. 3a,b).

**Fig. 4:**
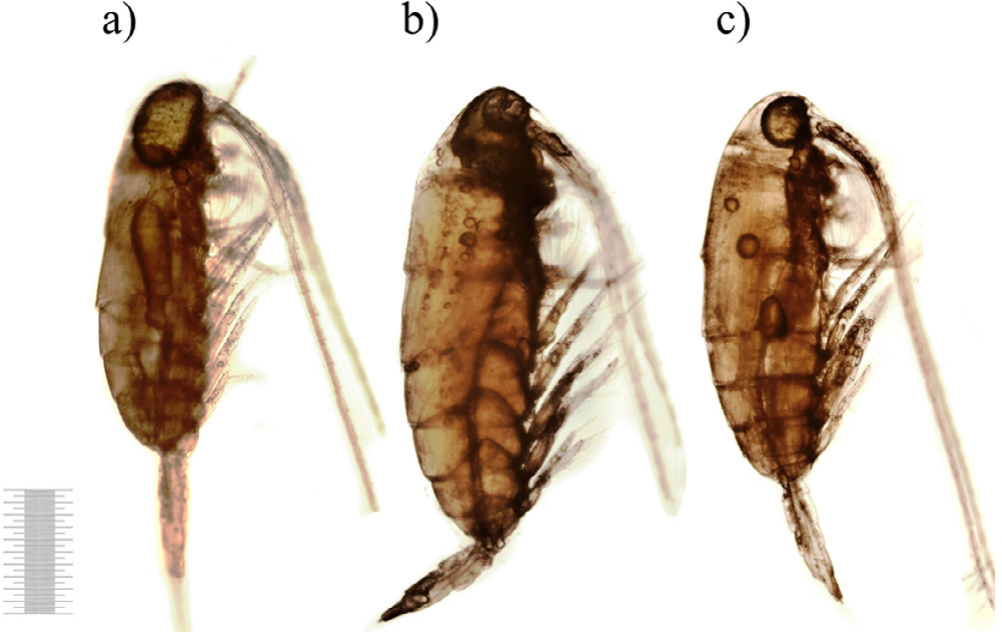
Decomposition of copepod carcasses on 1st day of the exposure (1 mm scale).

After 2 days of decomposition the external condition of carcasses that did not significantly differ between control and treatments is shown in Fig. 4. The color saturation did not change nonetheless the shade of some carcasses became darker. The external integument was intact, however, consistent with more detailed observations, insignificant gaps and voids appeared in separate areas of the carcasses (Fig. 4b,c). The oil sac area was significantly smaller comparing to the control. The oil sac was often spherical in shape and divided into separate smaller fragments (Figs. 4a,c).

**Fig. 5:**
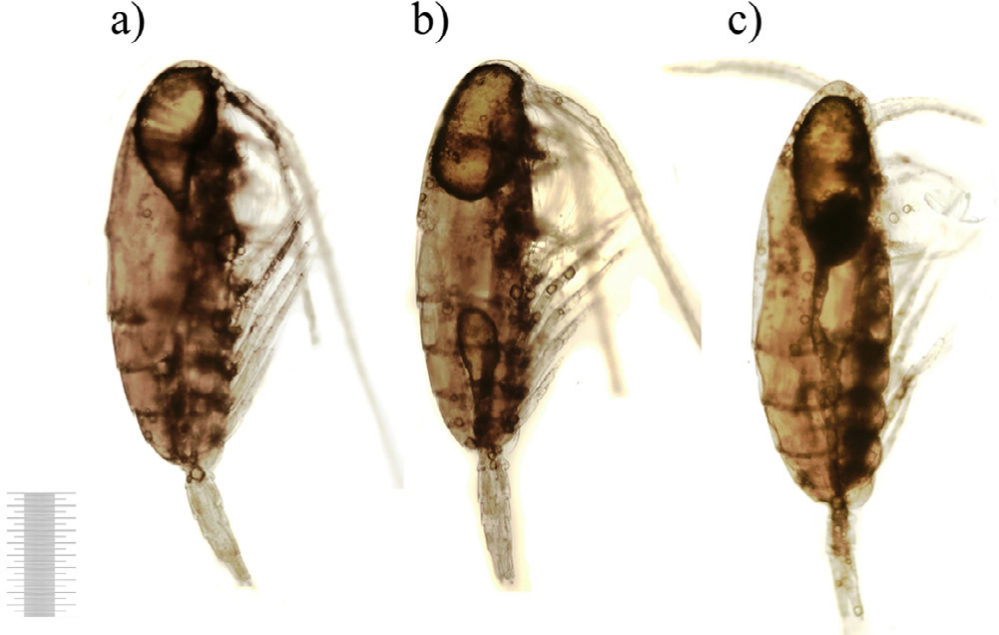
Copepod carcasses on 3rd day of the exposure (1 mm scale).

The carcasses presented in Fig. 5 remained in good condition with dark brown colored bodies, integument integrity and the presence of internal tissues. The large oil sac was mainly located in the cephalon area. However, the sample contained carcasses that seemed lighter and more transparent with obvious muscles detachments from carapace usually on the dorsal side of the body and in the abdominal region (Fig. 5c).

**Fig. 6:**
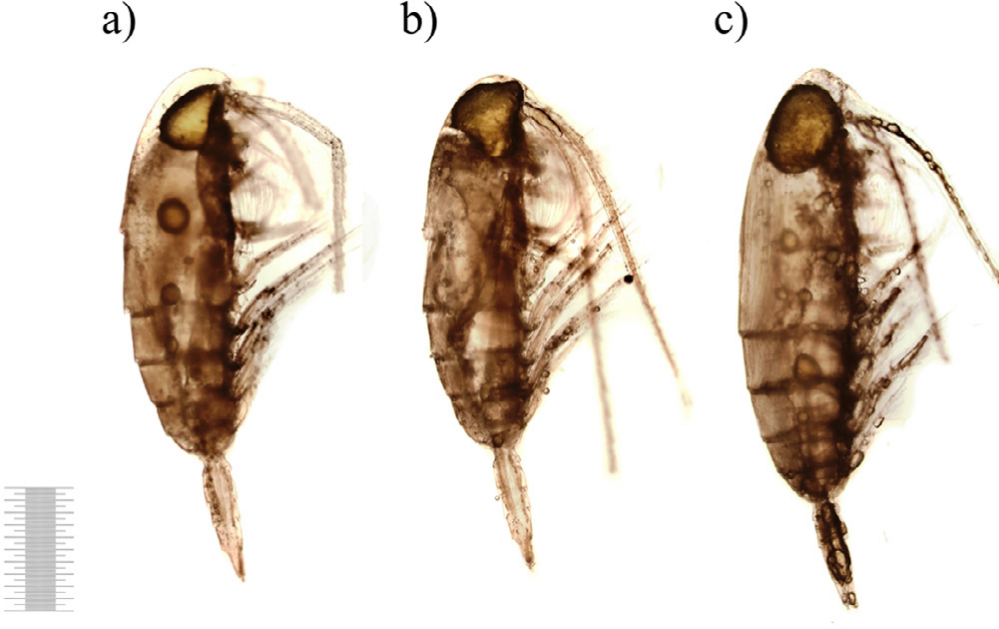
Copepod carcasses after 5 days of decomposition (1 mm scale).

After 5 days of decomposition, all carcasses were significantly paler than in the control sample (Fig. 6). The integument was resistant to decomposition but few ruptures of muscle fibers were observed. Tissue detachments and gaps occupied large areas, for example, in the cephalon area as shown in Fig. 6a. Along with one or more large oil sacs, smaller sacs appeared in atypical locations such as antennae or swimming legs (Fig. 6c).

**Fig. 7:**
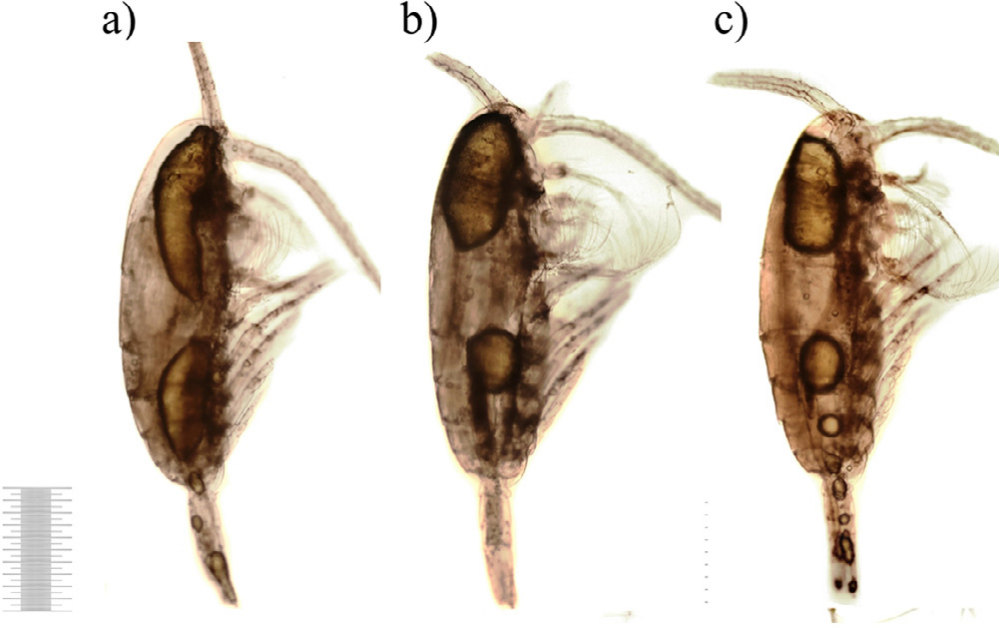
Copepod carcasses on 7th day of decomposition (1 mm scale).

The fraction of carcasses with obvious post-mortal changes increased on Day 7 (Fig. 7). Although the chitinous exoskeleton remained intact, almost all carcasses have partly lost internal tissues in the antennae and/or the abdomen (Fig. 7a,b). Copepods became more transparent. Deterioration of oil sacs did not generally differ from Day 5. Every oil sac was divided into two large and multiple small parts (Fig. 7).

**Fig. 8:**
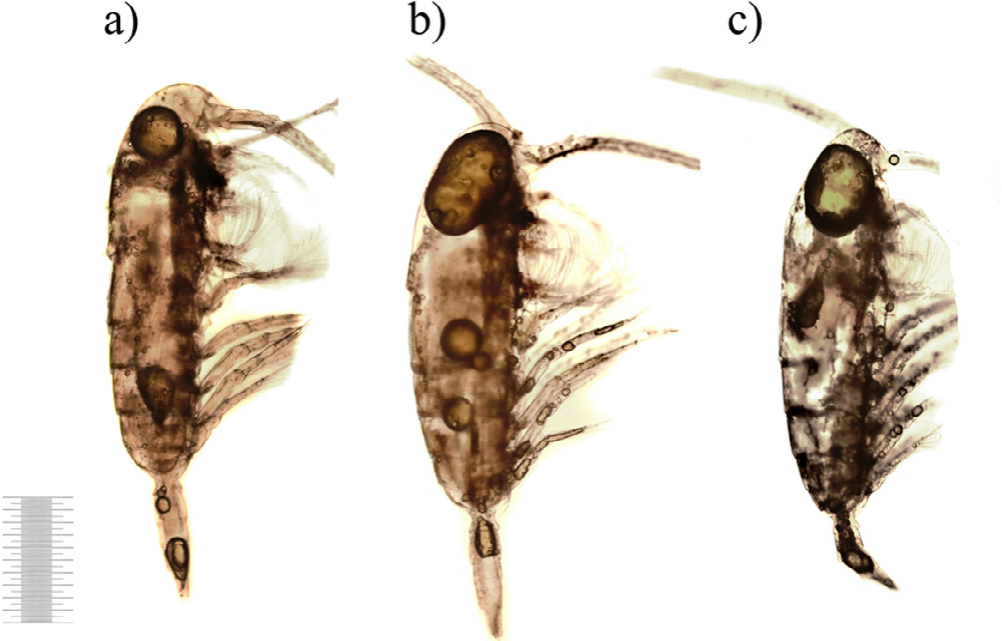
Copepod carcasses after 14 days of the exposure (1 mm scale).

After 14 days of the exposure, carapaces of most carcasses were still resistant to decomposition, and only rarely had cracks and fractures. Nevertheless, the carcasses could be classified into two main groups based on the level of decomposition. Some of the carcasses had significant loss of internal tissues, muscles detachments and ruptures but remained yellow-brown in color (Fig. 8a,b). While in the other group, the carcasses were translucent and lost more than half of the initial volume of their tissues which were noticeably macerated (Fig. 8c). Oil sacs of different sizes were found in most of the carcasses.

**Fig. 9:**
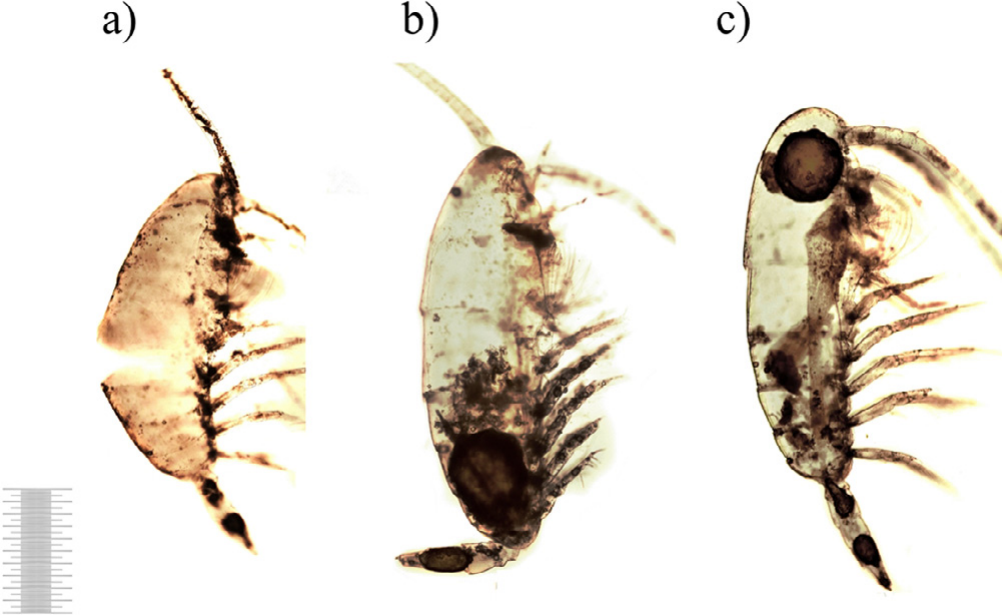
Decomposition of copepod carcasses on Day 21 (1 mm scale).

On the 21st day of the decomposition, 84% of the carcasses represented empty carapaces or fragments of carapaces (Fig. 9a) with trace amounts of internal tissues (Fig. 9b,c). However, copepod carcasses were not completely decomposed even after three weeks. The estimation of the total number of carcasses and, in some cases, the species determination was still possible. Oil sacs remained in some individuals (Fig. 9).

## 2 Experimental design, materials, and methods

The microcosm experiment was performed with seawater (salinity 31.45 psu) and zooplankton collected from the Kara Sea (near the Kara Strait). Zooplankton samples were collected by a vertical tow (0.8 m mouth diameter) with a 500 µm mesh net at upward speed of 0.6–0.8 m sec^‑^^1^. The zooplankton were dried, then frozen and stored in liquid nitrogen for later use. 25 bottles were filled with filtered through 0.45 µm membrane seawater to the final water volume of 250 ml. Bottles were incubated in thermostat for 21 days (referred to as experimental days 0, 1, 3, 5, 7, 14, 21) at a temperature ranged from −1.2 to −0.9 °C (mean −1.1 ± 0.1 °C). The temperature and salinity of the water in the experiment corresponded to the parameters of the natural habitat of copepods *Calanus* spp. [1]. 18 microcosms (3 frequencies for each experimental day 1–21) were enriched on Day 0 with zooplankton. Six others (one for each experimental day 1–21) received no manipulation and served as control. Water was sampled on Day 0 in order to determine the initial bacterial abundance and biomass of the seawater. Samplings of three experimental and one control microcosms were performed every experimental day at the same time. The first samplings were performed 24 h after zooplankton addition.

For the determination of the post mortem morphological changes of copepods 15 ml of water with at least 250 copepod carcasses were sampled and fixed with buffered formaldehyde (4% final concentration). The laboratory analysis was focused only on copepodites (CV) and the adults of this group, considering that the majority of copepods were calanoids. Copepod carcasses (mostly *Calanus* spp.) were visually analyzed and photographed under an inverted microscope Nikon Eclipse TS-100 equipped with digital camera Nikon D5100, 4×. We applied a method [2] based on visual evaluation of postmortem changes in every replicate of copepod carcasses. Visual changes and the most typical signs of decomposition of *Calanus* spp. carcasses were described for each experimental day.

The rest of the contents of each experimental microcosm were filtered through a 60 µm mesh net to collect zooplankton. The net content was rinsed twice with Milli-Q water. Excess water was removed with filter paper. Next, the zooplankton samples were placed in Petri dish with cover and frozen at −25 °C. Then samples were frozen for lyophilization (condenser temperature −85 °C, vacuum 1.0 mbar) for subsequent chemical analysis [3].

Water samples were fixed with 0.2 -µm prefiltered buffered formaldehyde (1% final concentration) for the determination of heterotrophic bacterial abundance. Fixed samples were filtered onto a nuclepore black filter (0.2 µm pore-size), stained with SYBR Green I (a final concentration of 10^–^^4^ of the commercial solution) and incubated 15 min in the dark [4,5]. Filters were completely dried, mounted on a glass slide with a drop of Immersion liquid (Leica, Type N) and covered with a coverslip. Slides were usually counted immediately but could be stored frozen at −20 °C for at least a few days. Observation and counts were made under an epifluorescent microscope (Leica DM 5000 B) at a magnification of × 1000. 20 fields and a total of >200 bacteria were counted for each filter. The bacterial wet biomass was estimated based on the individual cell volume using ImageScope Colour image analysis software. The bacterial biomass carbon was estimated based on bacterial cell volume, using the formula:

$$\text{fgC}\times {\text{cell}}^{-1}=133.754\times V^{0.438}$$

where fgC × cell^−1^ – carbon content (in femtograms) per bacterial cell, V – cell volume (µm^3^) [6]. In order to avoid contamination, clean gloves were used during the preparation as well as sampling period.

Statistical analyzes were performed in MS Excel and Statgraphics Plus Software.

## Appendix Supplementary materials

Image, application 1
